# Effect of Glenohumeral Joint Bone Morphology on Anterior Shoulder Instability: A Case-Control Study

**DOI:** 10.3390/jcm12154910

**Published:** 2023-07-26

**Authors:** Aybars Kıvrak, İbrahim Ulusoy

**Affiliations:** 1 Adana Avrupa Hospital, 01170 Adana, Turkey; aybarskivrak@gmail.com; 2 Department of Orthopedic Surgery, Selahhadin Eyyubi State Hospital, 21100 Diyarbakır, Turkey

**Keywords:** glenohumeral joint, bone morphology, shoulder instability, anterior shoulder dislocation

## Abstract

Purpose: Glenohumeral joint compatibility and bone morphology are among the most critical factors in shoulder stabilization. Our study investigated the effect of the bone morphological structure of the shoulder joint on anterior shoulder dislocation. Methods: In our study, people with a history of shoulder dislocation were selected as the patient group. In the control group, patients with shoulder MRIs for any reason and no history of shoulder dislocation were included. Those who have a fracture around the shoulder, a congenital deformity in the shoulder region, arthrosis of the shoulder, those whose MRI images cannot be measured, those with Hill-Sachs lesion, connective tissue diseases (such as Ehler Danlos), who are unsure of their diagnosis, or who have incomplete and incorrect suspicious information in their patient file have been excluded. In our retrospective case-control study, glenoid width, glenoid height, glenoid’s height-to-width ratio, glenoid’s depth, glenoid’s version, glenoid’s inclination, humerus radius of curvature, glenoid radius of curvature, and bony shoulder stability ratio were measured on MRI images of the patients. The sample size for each group was determined using a power analysis method. The intra-class coefficient (ICC) assessed interobserver and intraobserver reliability. Results: A total of 80 patients, 40 each in the control and patient groups, were included in the study. Glenoid width was measured as 24.27 ± 1.58 in the patient group, 25.61 ± 1.72 in the control group; glenoid height was as measured 36.49 ± 2.26 in the patient group, 36.74 ± 1.99 in the control group; height-to-width ratio was measured as 1.5 ± 0.08 in the patient group, 1.43 ± 0.05 in the control group; glenoid version was as measured −0.53 ± 1.17 in the patient group, −1.44 ± 1.1 in the control group; glenoid inclination was measured as 1.44 ± 3.93 patient group, 2.64 ± 3.81 in the control group; glenoid depth was measured as 1.69 ± 0.41 in the patient group, 2.12 ± 0.53 in the control group; humerus radius of curvature was measured as 29.70 ± 6.76 in the patient group, 24.98 ± 3.22 in the control group; glenoid axial radius of curvature was measured as 61.8 ± 13.52 in the patient group, 52.53 ± 15.69 in the control group; glenoid coronal radius of curvature was measured as 43.01 ± 7.47 in the patient group, 37.74 ± 6.89 in the control group; the bony shoulder stability ratio was measured as 0.35 ± 0.06 in the patient group and 0.44 ± 0.06 in the control group. In the statistical evaluation, the glenoid width (*p* < 0.001), the glenoid height/width ratio (*p* < 0.001), the glenoid version (*p* < 0.001), the depth of the glenoid cavity (*p* < 0.001), and the radius of curvature measurements of the humeral head (*p* < 0.001) and the glenoid (axial, *p* < 0.007; coronal, *p* < 0.001) were found to be significantly different. Glenoid height and inclination were similar in both groups. Conclusions: The detection of bone morphological features that constitute risk factors for shoulder dislocations plays an important role in preventing shoulder dislocations. In this way, it provides essential data on personalized rehabilitation programs and treatment selection for recurrent dislocations.

## 1. Introduction

Many risk factors have been described in the literature for shoulder dislocations. Many factors, such as age, gender, interest in contact sports, and occupational status, have been cited [1]. Some risk factors can be modified, and some can be described as non-modifiable. In addition, glenohumeral joint stability is dynamically and passively maintained. While the contraction of the muscles contributes to dynamic stabilization, the harmony and morphological structure of the glenohumeral joint are also effective in passive stabilization.

The stability ratio in shoulder biomechanics refers to the ratio between the maximum translational force that a shoulder can withstand and the corresponding concavity-compression force required for stabilization [2]. From this, it is concluded that the bone morphological structure of the shoulder joint may affect stability.

Few publications in the literature examine the bone morphological structure of the shoulder joint. A comprehensive evaluation has not been made in these publications. Our study investigated the effect of glenohumeral joint bone morphology on anterior shoulder instability by conducting a case-control study.

## 2. Materials and Methods

The research was conducted in compliance with the approval of the non-invasive clinical ethics committee of Muş Alparslan University (Approval No. E-10879717-050.01.04-5500). Individuals who applied to the orthopedic outpatient clinic in a single center between 2017 and 2020 were included in the study. The study was designed as a retrospective case-control study. In our study, patients between the ages of 18 and 65 who were diagnosed by an orthopedic specialist or had a history of anterior shoulder dislocation with the help of radiological images were included in the case group. Those who have a fracture around the shoulder, a congenital deformity in the shoulder region, arthrosis of the shoulder, a bony bankart lesion, those whose MRI images cannot be measured, those with a Hill-Sachs lesion, connective tissue diseases (such as Ehler Danlos), who are unsure of their diagnosis, or who have incomplete and incorrect suspicious information in their patient file have been excluded. Patients who underwent an MRI due to shoulder pain and who had tendinitis or no pathology were included in the control group. Exclusion criteria in the case group were also applied in the control group. Individuals were included in the study by performing computer-assisted randomization (www.randomizer.org). The demographic characteristics of the individuals included in the study were extracted.

### 2.1. Outcome Measure

The imaging of all patients was done with a 1.5 Tesla MRI. Images include coronal, axial, and sagittal slices as standard.

The widest points between the outer edges of the corners of the glenoid bone were measured in both the axial and coronal planes to determine the width and height of the glenoid.

The glenoid version was measured by the method described by Tetrault et al. [3]. Two lines are required for this measurement. The first line is drawn along the glenoid axis. The other line is the axis of the supraspinatus fossa. To determine the glenoid version, the angle formed between the glenoid surface and the supraspinatus fossa axis is measured and then subtracted from 90 degrees. A positive angle defines anteversion, and a negative angle determines retroversion.

Glenoid inclination was measured as described by Maurer et al. [4]. In the coronal oblique view, the deepest point of the supraspinatus fossa was determined. A line was drawn from the scapula’s body to the supraspinatus’s deepest point. Another line was pulled from the upper and lower borders of the glenoid fossa. As in the glenoid version calculation, the resulting value was subtracted from 90. The positive angle reflects the caudal inclination, and the negative angle reflects the cranial inclination.

Glenoid concavity depth was measured as described in another publication [2]. In the axial section, a line connecting both apexes of the concavity of the glenoid is drawn. The deepest point of the concavity of the glenoid was then measured.

**Figure 1:**
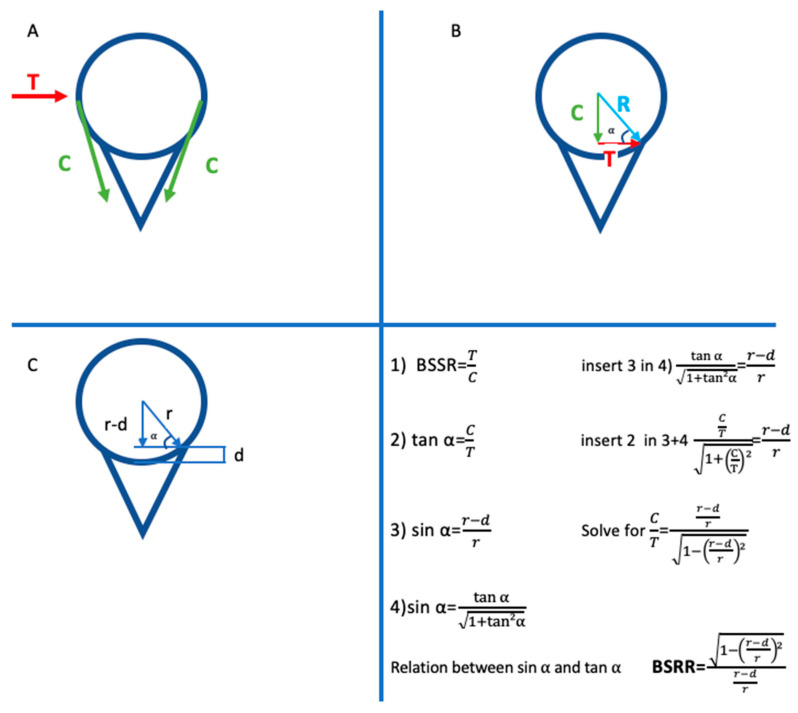
“(**A**) The ball-and-socket configuration of a glenohumeral joint with depicted translational force (T) and compressive force (C). (**B**) The compressive force vector (C) and the translational force vector (T) form a right triangle, with the resulting force vector as the hypotenuse (R). (**C**) An identical right triangle is described by geometrical entities of the ball-and-socket configuration, including the radius (r) and the concavity depth (d) or combinations thereof. Mathematical considerations based on Pythagorean trigonometric identities put the right triangle of the force vectors about the right triangle of the geometrical entities to determine the bony shoulder stability ratio (BSSR), which is the ratio for the maximal translational force (T) against which a shoulder can be stabilized by a given concavity-compression force (C)” Reprinted with permission from ref. [2]. Copyright 2015 Arthroscopy Association of North America.

Glenoid superior-inferior and anterior-posterior radius of curvature (ROC) were measured in coronal and axial sections. The measurement was made by drawing a circle that fits on the glenoid borders. Finally, the radius of the curvature of the humerus was obtained by drawing the circle with the largest diameter in which the humeral head entirely fits in the axial sections. Using geometric measurements, a formula called bony shoulder stability ratio (BSSR) has been developed to calculate the stability ratio in the shoulder joint [2]. This measurement used the glenoid depth and the radius of the humeral head (Figure 1). Two orthopedic specialists in the T1 sequence made all measurements.

### 2.2. Reliability

To establish interobserver and intraobserver reliability, some assessments have been made. All measurements were made twice, with an interval of two weeks, by the clinician who made the measurement. In addition, all measurements were made again by an independent orthopedic specialist to evaluate interobserver reliability. The intra-class coefficient (ICC) assessed interobserver and intraobserver reliability. The ICC is a numerical value ranging from 0 to 1, where values less than 0.5 indicate poor reliability, values between 0.5 and 0.75 indicate moderate reliability, values between 0.75 and 0.9 indicate good reliability, and values above 0.9 indicate excellent reliability [5].

### 2.3. Statistical Analysis

Statistical analyses were performed using the statistical package for the social sciences (IBM SPSS 28.0.1.0; Corp., Armonk, NY, USA). The normality of the variables was assessed using visual methods (histograms, probability plots) and analytical tests (Kolmogorov–Smirnov/Shapiro–Wilk’s test). For normally distributed variables, descriptive statistics were presented as means and standard deviations. The Student’s *t*-test was employed to compare parameters, and a *p*-value less than 0.05 was considered statistically significant. The sample size for each group was determined using a power analysis method. G*Power Version 3.1.9.6 (Heinrich-Heine-Universität Düsseldorf, Düsseldorf, Germany) was used in the power analysis. The alpha value was determined to be 0.05, and the power was 0.95. While determining the effect size, the study data of Peltz et al. (superior/inferior radius of curvature, anterior/posterior radius of curvature) were used [6].

## 3. Results

When the data between 2017 and 2020 were scanned, 173 patients were found to meet the criteria, considering the inclusion and exclusion criteria. Forty patients were randomly selected. When we analyzed the demographic data of the patients and the control group, the mean age of the patient group was 33.57 ± 14.39, and the mean age of the control group was 34.25 ± 13.42. There were 33 males and 7 females in the patient group and 29 males and 11 females in the control group. The right shoulder of 31 patients, the left shoulder of 9 patients in the patient group, the right shoulder of 26 patients in the control group, and the left shoulder of 14 patients were evaluated (Table 1). In the statistical evaluation, no significant difference was found between the age, gender, and side of the study group and the patient group.

In the statistical evaluation, the glenoid’s width, the glenoid’s height/width ratio, the glenoid version, the depth of the glenoid cavity, and the ROC measurements of the humeral head and the glenoid were found to be significantly different. Within the patient group, the glenoid width was determined as 24.27 ± 1.58, while in the control group, it was 25.61 ± 1.72. The patient group exhibited a glenoid height of 36.49 ± 2.26, whereas the control group had a height of 36.74 ± 1.99. The height-to-width ratio within the patient group was 1.5 ± 0.08, compared to 1.43 ± 0.05 in the control group. Regarding the glenoid version, the patient group had a value of −0.53 ± 1.17, while the control group had −1.44 ± 1.1. The glenoid inclination in the patient group was 1.44 ± 3.93, whereas it was 2.64 ± 3.81 in the control group. The glenoid depth within the patient group measured 1.69 ± 0.4, while the control group measured 2.12 ± 0.53. The Humerus ROC in the patient group was found to be 29.70 ± 6.76, whereas in the control group, it was 24.98 ± 3.22. Additionally, the glenoid axial ROC within the patient group was 61.8 ± 3.52, compared to 52.53 ± 15.69 in the control group. Glenoid height and inclination were similar in both groups. Additionally, statistically significant differences were found between the patient and control groups in terms of glenoid width, height-to-width ratio, glenoid version, glenoid depth, humerus ROC, glenoid axial ROC, glenoid coronal ROC, and BSSR measurements (Table 2). Interobserver and intraobserver reliability were determined to be excellent (Table 3).

## 4. Discussion

Our study determined bone morphological features associated with anterior shoulder dislocation. A high glenoid height-to-width ratio, high anteversion of the glenoid, low glenoid depth, high ROC value of the humerus and glenoid (i.e., flat humeral head and glenoid, loss of sphericity), and low BSSR value predispose to anterior shoulder dislocation. To our knowledge, no published research has investigated these anatomical morphological features in the same patient group.

In a healthy shoulder, perfect harmony and stability are required during movement. Soft tissue and bone structures achieve this harmony and stability passively or actively. Active-dynamic stabilization occurs with active contraction of the muscles involved in the movement of the shoulder joint. On the other hand, passive stabilization is provided by the bone anatomical structure, cartilage structure, labrum, and passive tension of the muscles and ligaments. The glenoid and humeral head structures are vital to this passive stabilization.

Studies in the literature have found a relationship between the shape of the glenoid and instability. Taller and thinner glenoids have been found to pose a risk for shoulder dislocation. A prospective cohort study of 714 young athletes determined that tall and thin glenoids were more at risk [7]. Another study determined that the glenoid height-to-width ratio poses a risk for anterior shoulder dislocation [8]. In our study, similar data were obtained from the literature. In the patient group, it was determined that the glenoid structure was narrower and the height-to-width ratio was higher. The narrow glenoid, which provides less contact surface, can explain the relationship between glenoid width and anterior shoulder dislocation. In a study, the mean glenoid track width was found to be 83% of the glenoid width. This percentage changes with shoulder movements [9]. However, as a result, when the width of the glenoid narrows, there is a risk of dislocation as the range of motion for the humeral head will be narrowed.

The effect of the glenoid version on the stability of the shoulder joint was first discussed in the investigation of the effect of retroversion of the glenoid component on posterior instability in shoulder arthroplasty [10]. However, static centralization and translation of the humeral head depending on the forces applied to the shoulder have begun to be investigated. A cadaver study determined that in the glenoid version between −5° and +5°, each 1° increase (increased anteversion of the glenoid) reduces the energy required for anterior shoulder dislocation by 10% and the force by 8% [11]. In addition, the same article states that for every 5° increase in glenoid retroversion, the energy required to create anterior dislocation increases by 50%. In another retrospective study, the MRI images of 128 patients with anterior shoulder dislocation were compared with the MRI images of a control group of 130 people. As a result, the glenoid version degree was higher in the group with anterior shoulder dislocation [12]. In our study, similar to the literature, it was found that the risk of anterior shoulder dislocation was higher in glenoids with high anteversion.

Very few publications in the literature examine the relationship between glenoid inclination and anterior shoulder dislocation. In addition, many publications are concerned with the relationship between glenoid inclination and rotator cuff tears. It has been emphasized in many publications that superior glenoid inclination is associated with rotator cuff tears [13,14,15]. A retrospective study by Hohman et al. found a significant relationship between anterior shoulder dislocation and inferior inclination [12]. In anterior shoulder dislocations with high inferior inclination, It is assumed that with the reduction of inferior humeral translation, the shoulder is more stable and dislocations are less common [12]. However, it was emphasized that biomechanical studies should support this assumption.

Net forces acting on the glenohumeral joint prevent dislocation of the shoulder joint. Concavity compression applied by the humeral head to the glenoid fossa also plays an essential role in stabilizing the shoulder [16]. Decreased glenoid depth results in a loss of concavity in the glenoid. This can reduce the compression applied by the rotator cuff, which is one of the primary stabilizers of the shoulder [17]. The BSSR measurement found by Moroder et al. is a calculation method that only considers bone structure [2]. BSSR values are directly and linearly related to the stabilization ratio at the shoulder [18]. This calculation quantifies the concavity-compression effect. In this respect, it is essential for evaluating glenohumeral joint bone morphology. Ernstbrunner et al. confirmed the validity of the BSSR in their biomechanical study [19]. In the studies performed, the BSSR value was low in anterior shoulder dislocations [2,17]. Similar results were obtained in our study.

MRI and CT are commonly used methods to evaluate the shoulder joint. While MRI is mainly used to assess soft tissue (such as the labrum, capsule, and rotator cuff), CT is used more frequently in pathologies of bone tissue. In the literature, morphological measurements of the glenohumeral joint were made with CT in some studies and with MR in some studies [6,12,20,21]. In our study, measurements were made with an MRI. We think that if the same patients were evaluated with CT, they would give similar results.

There are some limitations to our study. Making the measurements with the orthopedic specialist can be considered a limitation. In addition, the contralateral shoulder images of individuals in the control and patient groups could not be examined. When we look at the studies in the literature, it is seen that there is no difference in the measurements of both shoulders belonging to the same individual [22,23]. Although patients with bony Bankart and Hill-Sach lesions were excluded from the study, the contralateral shoulder imaging evaluation may increase the study’s power. Power analysis is less preferred in retrospective studies than in prospective studies. However, in retrospective evaluation, you can access as many patient files as you want, as long as the hospital’s archive allows. A power analysis was performed to give an idea of the sample size we will study. To help reduce bias and the margin of error, 40 patients were selected by randomization. Although soft tissues are not considered in the measurements, measurements on MR imaging instead of CT can be considered a limitation. Including patients with one shoulder dislocation in our study is another limitation. Although it was stated in a study that recurrence occurred in 79.7% of patients after the first dislocation, it is not certain that there is instability in those with a history of one dislocation [24]. The retrospective nature of our research can be considered a limitation.

## 5. Conclusions

Glenoid-humeral head alignment in the shoulder is crucial to stabilizing the shoulder. Many modifiable and non-modifiable risk factors for shoulder dislocation have been described in the literature. Our study demonstrated that the bone morphological structure of the shoulder might also pose a risk of anterior shoulder dislocation. This situation can be evaluated with other risk factors, and personalized rehabilitation programs can be developed in the community. The risk of anterior shoulder dislocation can be reduced in athletes and normal individuals.

## Figures and Tables

**Table 1:** Demographic characteristics of the patient and control groups.

	Patient Group	Control Group	*p* Value
Age (Mean ± Standard Deviation)	33.57 ± 14.39	34.25 ± 13.42	0.829
Gender (Male/Female)	33/7	29/11	0.284
Side (Right/Left)	31/9	26/14	0.217

**Table 2:** Bone morphological measurements.

	Patient Group	Control Group	*p* Value
Glenoid widht	24.27 ± 1.58	25.61 ± 1.72	<0.001
Glenoid height	36.49 ± 2.26	36.74 ± 1.99	0.601
Height-to-width ratio	1.5 ± 0.08	1.43 ± 0.05	<0.001
Glenoid version	−0.53 ± 1.17	−1.44 ± 1.1	<0.001
Glenoid inclination	1.44 ± 3.93	2.64 ± 3.81	0.17
Glenoid depth	1.69 ± 0.41	2.12 ± 0.53	<0.001
Humerus ROC	29.70 ± 6.76	24.98 ± 3.22	<0.001
Glenoid axial ROC	61.8 ± 13.52	52.53 ± 15.69	0.007
Glenoid coronal ROC	43.01 ± 7.47	37.74 ± 6.89	<0.001
BSSR	0.35 ± 0.06	0.44 ± 0.06	<0.001

**Table 3:** Interobserver and intraobserver reliability of the morphological bone measurements.

	Interobserver		Intraobserver	
	ICC (95%CI)	*p* Value	ICC (95%CI)	*p* Value
Glenoid widht	0.954 (0.928–0.970)	<0.001	0.983 (0.973–0.989)	<0.001
Glenoid height	0.939 (0.905–0.961)	<0.001	0.986 (0.978–0.991)	<0.001
Glenoid version	0.933 (0.896–0.957)	<0.001	0.980 (0.970–0.987)	<0.001
Glenoid inclination	0.969 (0.951–0.980)	<0.001	0.989 (0.983–0.993)	<0.001
Glenoid depth	0.907 (0.855–0.940)	<0.001	0.943 (0.912–0.964)	<0.001
Humerus ROC	0.987 (0.980–0.992)	<0.001	0.997 (0.996–0.998)	<0.001
Glenoid axial ROC	0.945 (0.914–0.964)	<0.001	0.988 (0.981–0.992)	<0.001
Glenoid coronal ROC	0.944 (0.913–0.964)	<0.001	0.974 (0.960–0.984)	<0.001

## Data Availability

The datasets used and analyzed during the current study are available from the corresponding author on reasonable request.
